# Vitamin D Status Determines Metformin Action on Gonadotropin Levels in Postmenopausal Women with Subclinical Hyperthyroidism

**DOI:** 10.3390/pharmaceutics17040442

**Published:** 2025-03-30

**Authors:** Robert Krysiak, Karolina Kowalcze, Witold Szkróbka, Bogusław Okopień

**Affiliations:** 1 Department of Internal Medicine and Clinical Pharmacology, Medical University of Silesia, Medyków 18, 40-752 Katowice, Poland; wszkrobka@sum.edu.pl (W.S.); bokopien@sum.edu.pl (B.O.); 2 Department of Pediatrics in Bytom, Faculty of Health Sciences in Katowice, Medical University of Silesia, Stefana Batorego 15, 41-902 Bytom, Poland; kkowalcze@sum.edu.pl; 3 Department of Pathophysiology, Faculty of Medicine, Academy of Silesia, Rolna 43, 40-555 Katowice, Poland

**Keywords:** gonadotropic cells, hyperthyroidism, insulin resistance, metformin, postmenopause, vitamin D status

## Abstract

**Background/Objectives:** The gonadotropin-lowering effects of metformin were found to be more pronounced in the case of coexisting hyperthyroidism and absent in patients with hypovitaminosis D. Thus, the aim of the current study was to determine whether vitamin D status determines pituitary effects of metformin in individuals with thyroid hyperfunction and elevated gonadotropin levels. **Methods:** This prospective cohort study included three matched groups of postmenopausal women with hyperthyroidism and prediabetes: women with 25-hydroxyvitamin D levels between 50 and 75 nmol/L (uncompensated vitamin D insufficiency), women with 25-hydroxyvitamin D levels between 75 and 150 nmol/L receiving exogenous calciferol due to previously diagnosed vitamin D deficiency/insufficiency (compensated vitamin D deficiency/insufficiency), and calciferol-naïve subjects with 25-hydroxyvitamin D levels between 75 and 150 nmol/L (the control group). Over the entire study period (six months), all the women were treated with metformin. At the beginning and at the end of this study, we determined 25-hydroxyvitamin D, glucose homeostasis markers, gonadotropins, estradiol, progesterone, TSH, free thyroid hormones, prolactin, ACTH, and IGF-1. **Results:** Before metformin treatment, except for the 25-hydroxyvitamin D levels, there were no between-group differences in the investigated markers. In all the study groups, metformin reduced plasma glucose, HOMA1-IR, glycated hemoglobin, and FSH, but these effects were more pronounced in both groups of women with normal vitamin D status than in women with uncompensated vitamin D insufficiency. The decrease in LH concentration was observed only in patients with compensated vitamin D deficiency/insufficiency and in the control group. There were no differences between the baseline and follow-up levels of the remaining hormones. The impact of metformin on gonadotropin concentrations positively correlated with their baseline values, free thyroid hormone levels, 25-hydroxyvitamin D levels, and metformin-induced changes in HOMA1-IR. **Conclusions:** Our findings suggest that low vitamin D status impairs the gonadotropin-lowering effects of metformin in individuals with hyperthyroidism.

## 1. Introduction

It is well documented that metformin inhibits the secretory function of at least three types of anterior pituitary cells: thyrotropic [1,2], lactrotropic [3,4], and gonadotropic [5,6,7,8] cells in states associated with their overactivity. The impact on gonadotropin secretion was observed in both women and men, independently of the reason for gonadotropin excess. The drug reduced luteinizing hormone (LH) in females with polycystic ovary syndrome [6], follicle-stimulating hormone (FSH) in women after menopause [7], and both FSH and LH in males with hypergonadotropic hypogonadism [8]. To date, three factors were found to determine metformin action on the hypothalamic–pituitary–gonadal axis. Firstly, the degree of reduction in gonadotropin levels depends on the degree of their hypersecretion. Metformin does not influence gonadotropin concentrations in individuals with FSH and LH levels within the reference range [8]. Treatment-induced changes in LH levels were more pronounced in women with polycystic ovary syndrome than the changes in FSH [5,6], while the opposite relationship was observed in postmenopausal women [7]. This may be explained by the fact that the LH to FSH ratio in the follicular phase of the cycle is usually higher in women with polycystic ovary syndrome than in their peers without ovarian disease [9], while low ovarian reserve, observed in postmenopausal women, is manifested by a more pronounced increase in FSH than LH [10]. Secondly, metformin action on gonadotropins was found to be modulated by comedications. Letrozole enhanced this action in women with polycystic ovary syndrome [11], while potentiating effects of statins [12,13] and myo-inositol [14] were reported in young women with polycystic ovary syndrome and in women after menopause. Lastly, the gonadotropin-lowering effect of metformin seems to be modulated by comorbidities. The strength of metformin action was attenuated by coexisting autoimmune thyroiditis [15] and hypovitaminosis D [16], while the opposite effect was exerted by coexisting hyperthyroidism [17].

The modulatory effects of low vitamin D status and hyperthyroidism may be clinically relevant because of their associations with type 2 diabetes and other insulin-resistant states. The pooled prevalence of vitamin D deficiency among patients with type 2 diabetes was estimated at 64.2%, and the risk was particularly high in individuals with poor glycemia control [18]. The association between low 25-hydroxyvitamin D and incident diabetes was observed even after adjusting for several relevant potential confounders [19]. Moreover, there was an inverse association between 25-hydroxvitamin D concentrations and the risk of metabolic syndrome in general adult populations [20]. In turn, thyroid hyperfunction in patients with diabetes has been estimated at 1.0%, occurring more frequently than in the general population in the Whickham survey (0.3%) [21]. The cumulative incidence of type 2 diabetes in patients with thyroid disease was higher than in individuals with normal thyroid function [22]. Moreover, treatment of thyroid hyperfunction is not obligatory or even not recommended in patients with thyroid-stimulating hormone (TSH) concentration in the range between 0.1 and 0.39 mU/L [23]. Lastly, low circulating 25-hydroxyvitamin D levels predispose to Graves’ disease [24], which is by far the most common cause of hyperthyroidism [25].

These findings suggest that many patients with mild forms of hyperthyroidism did not receive specific treatment, despite being treated with metformin, and a significant proportion of them may be characterized by low vitamin D status. Middle-aged and elderly individuals may be at the highest risk because the prevalence of hyperthyroidism, insulin resistance-associated disorders, and calciferol deficiency/insufficiency increases with age [26,27,28]. Thus, the aim of our study was to investigate a putative interaction between hyperthyroidism and low vitamin D status on pituitary effects of metformin in postmenopausal women.

## 2. Materials and Methods

This study has been performed in a way that is consistent with the ethical standards articulated in the 1964 Declaration of Helsinki and its subsequent amendments. Ethics approval was obtained from the local review board. All the patients gave their written informed consent after learning of the risks, benefits, and other important information relevant to this study. Because of its nature, this study was not required to be registered in a public trial registry.

### 2.1. Study Population

**Figure 1:**
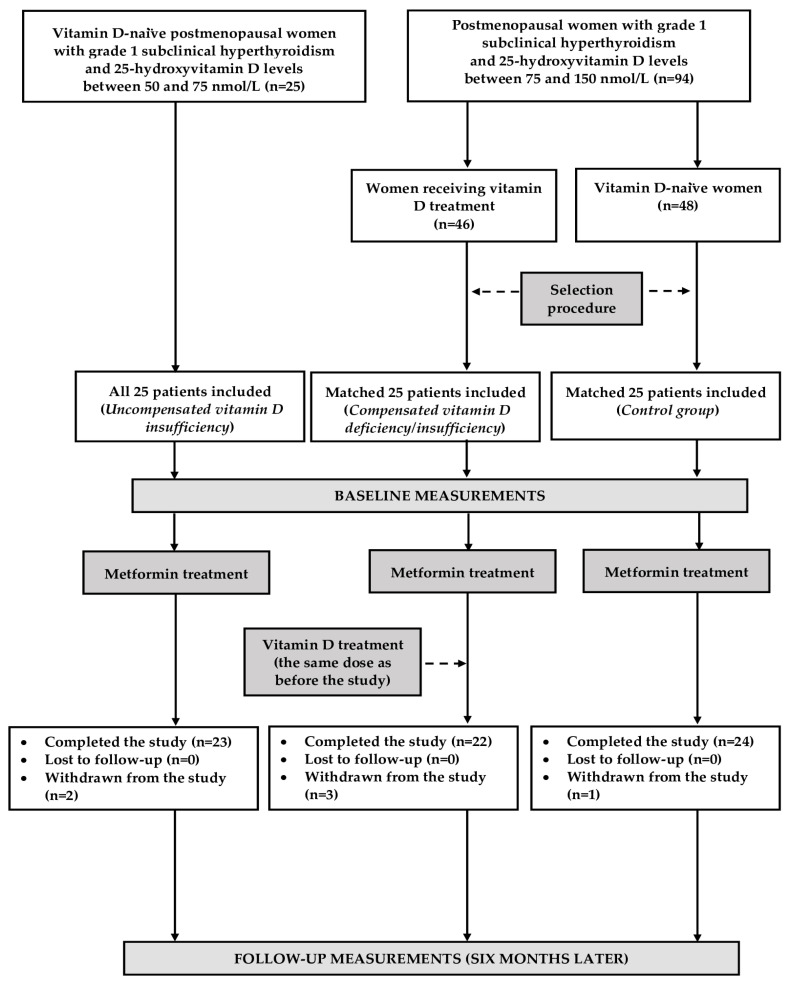
The flow of patients through this study.

The participants of this prospective matched cohort study were recruited among postmenopausal women with grade 1 subclinical hyperthyroidism and prediabetes. Only women complying with the lifestyle modification for at least 12 weeks were considered for enrollment. Postmenopause was defined as absence of a menstrual cycle for 12 or more months, coexisting with FSH concentrations exceeding 30 IU/L, and estradiol levels less than 110 pmol/L. Prediabetes was diagnosed on the basis of criteria of the American Diabetes Association [29]. Grade 1 hyperthyroidism was diagnosed if concentrations of thyroid-stimulating hormone (TSH) was in the range between 0.1 and 0.39 mU/L, but levels of free thyroxine and free triiodothyronine were within the normal ranges (between 10.5 and 21.5 pmol/L and 2.1 and 6.5 pmol/L, respectively) [30]. Based on 25-hydroxyvitamin D levels [31] and calciferol supplementation, the participants were divided into three groups, each consisting of 25 women. Twenty women were needed in each study group to obtain a power of 80% for a two-sided test with the significance level at 5%. However, to compensate for possible losses during the follow-up period, the sample size was increased by 25%. The first group included women with 25-hydroxyvitamin D levels between 50 and 75 nmol/L not receiving vitamin D supplementation (patients with uncompensated vitamin D insufficiency). The remaining two groups included women with normal vitamin D status (25-hydroxyvitamin D levels between 75 and 150 nmol/L). Owing to vitamin D deficiency diagnosed in the past, the patients assigned to the second received vitamin D supplementation for at least 4 months in individually constant doses (50–100 μg daily) (women with compensated vitamin D deficiency/insufficiency). In turn, group 3, which was the control group, included women with normal vitamin D homeostasis not receiving calciferol supplementation. Because of ethical concerns, vitamin D-naïve women with a 25-hydroxyvitamin D concentration below 50 nmol/L were not considered eligible for this study. All the eligible patients with vitamin D insufficiency were included in this study. The remaining two groups were selected from larger groups of women meeting the respective inclusion criteria and who agreed to participate (Figure 1). The chosen selection algorithm was aimed at matching the study groups for age, insulin sensitivity, and concentrations of FSH and LH. In order to minimize the impact of seasonal fluctuations in vitamin D status and seasonal confounds [32], similar numbers of individuals were recruited in each season: spring (18 women: 7 with uncompensated vitamin D insufficiency, 5 with compensated vitamin D deficiency/insufficiency, and 6 in the control group), summer (20 women: 6 with uncompensated vitamin D insufficiency, 7 with compensated vitamin D deficiency/insufficiency, and 7 in the control group), autumn (19 women: 6 with uncompensated vitamin D insufficiency, 7 with compensated vitamin D deficiency/insufficiency, and 6 in the control group), and winter (18 women: 6 in each group).

The exclusion criteria were as follows: other endocrine disorders, chronic inflammatory or autoimmune disorders, cardiovascular disease (except for grade 1 hypertension), impaired renal or liver function, malabsorption syndrome, or other serious disorders. We also excluded women on hormone replacement therapy or treated within the last six months with drugs found to affect insulin sensitivity, activity of the hypothalamic–pituitary–ovarian axis, or levels of other hormones.

### 2.2. Study Design

For the following six months, all the women received metformin and continued to follow the lifestyle modification. The drug was administered per os and swallowed without splitting, chewing, or crushing the tablets. The starting dose of metformin (500 mg twice a day) was administered in the first week of treatment. Then, the dose was gradually increased to 1000 mg three times a day (3 g daily), administered for the remaining period of time with or shortly after meals. Medication adherence was measured by counting the number of residual tables. A patient was considered adherent if 80–110% of the prescribed medication was used. Women with compensated vitamin D deficiency/insufficiency continued calciferol supplementation at the same dose as before. During the course of this study, new treatments were allowed if they were short term (below 10 days), not mentioned in the exclusion criteria, and terminated at least six months before the final visit. Participation in this study was discontinued in the case of consent withdrawal, serious adverse effects, sudden hospitalization, and changes in pharmacotherapy (except for mentioned above).

### 2.3. Laboratory Assays

All the measurements were performed on the first study day (before the first metformin dose) and after six months of metformin therapy. Blood samples were drawn from the right antecubital vein in the outpatient room by trained phlebotomists. All the venipunctures were performed in the morning following a 12 h fast. The assays were carried out in duplicate (in order to minimize analytical errors) by a technician who was not familiar with the study details. The plasma levels of glucose and whole blood content of glycated hemoglobin (HbA_1c_) were measured using the multi-analyzer COBAS Integra 400 Plus (Roche Diagnostics, Basel, Switzerland). The plasma levels of 25-hydroxyvitamin D, insulin, gonadotropins, estradiol, progesterone, TSH, free thyroxine, free triiodothyronine, and prolactin were measured by direct chemiluminescence using acridinium ester technology (ADVIA Centaur XP Immunoassay System, Siemens Healthcare Diagnostics, Munich, Germany). The glucose and insulin concentrations were used to calculate the homeostatic model assessment 1 of insulin resistance (HOMA1-IR), a surrogate marker of insulin sensitivity [33]. The remaining markers, adrenocorticotropic hormone (ACTH) and insulin-like growth factor-1 (IGF-1), were measured using solid-phase, enzyme-labeled, chemiluminescent immunoassays on a Siemens Immulite analyzer (Munich, Germany). The assay sensitivities were as follows: 8 nmol/L for 25-hydroxyvitamin D, 0.6 mg/dL for glucose, 0.62 mU/L for insulin, 3.9% for HbA_1c_, 0.3 U/L for FSH, 0.1 U/L for LH, 29 pmol/L for estradiol, 0.6 nmol/L for progesterone, 0.008 mU/L for TSH, 1.3 pmol/L for free thyroxine, 0.3 pmol/L for free triiodothyronine, 0.6 ng/mL for prolactin, 9 pg/mL for ACTH, and 14 ng/mL for IGF-1. The intra-assay and inter-assay coefficients of variations were 4.6% and 7.3% for 25-hydroxyvitamin D, 1.6% and 2.0% for glucose, 5.0% and 6.2% for insulin, 2.0% and 2.8% for HbA_1c_, 3.5% and 5.8% for FSH, 2.3% and 3.1% for LH, 4.0% and 6.5 for estradiol, 5.5% and 6.9% for progesterone, 2.0% and 3.4% for TSH, 3.0% and 5.0% for free thyroxine, 3.1% and 4.6% for free triiodothyronine, 2.9% and 4.7% for prolactin, 3.8% and 7.8% for ACTH, and 4.7% and 5.6% for IGF-1.

### 2.4. Statistical Analysis

The raw data were subjected to log transformation to achieve normality before further analyses, and then back-transformed. Between-group comparisons were analyzed using one-way analysis of variance, with subsequent pairwise testing using the Bonferroni method. Student’s paired *t*-test was used to identify differences between the means of variables in the same study group. The categorical variables were compared using the chi-square test. The strength and direction of the associations between the outcome variables were measured using Pearson’s correlation coefficient. Statistical significance was assumed if the *p*-values were less than 0.05.

## 3. Results

### 3.1. The Course of This Study

Six patients prematurely terminated this study. Adverse gastrointestinal effects were reported in four metformin-treated patients: one woman with uncompensated vitamin D insufficiency, two patients with compensated vitamin D deficiency/insufficiency, and one individual from the control group. Two other patients, one with uncompensated vitamin D insufficiency and one with compensated vitamin D deficiency/insufficiency, were withdrawn because of, respectively, acute hospitalization (due to pneumonia) and the initiation of a new treatment (atorvastatin). Thus, the final analysis included 69 patients who completed this study and adhered to the treatment recommendations. A post hoc power analysis showed that this study was sufficiently powered. In the group of women with compensated vitamin D deficiency/insufficiency, the mean intake of vitamin D (excluding its intake with food) was 69.8 ± 15.0 µg.

### 3.2. Baseline Conditions

Before metformin treatment, there were no differences between the study groups in age, reasons for hyperthyroidism, smoking habits, body mass index, values of blood pressure, and daily intake of calciferol with food. The only laboratory variable differing between the study groups was 25-hydroxyvitamin D concentration (Table 1).

### 3.3. The Effect of Metformin on the Investigated Variables

**Figure 2:**
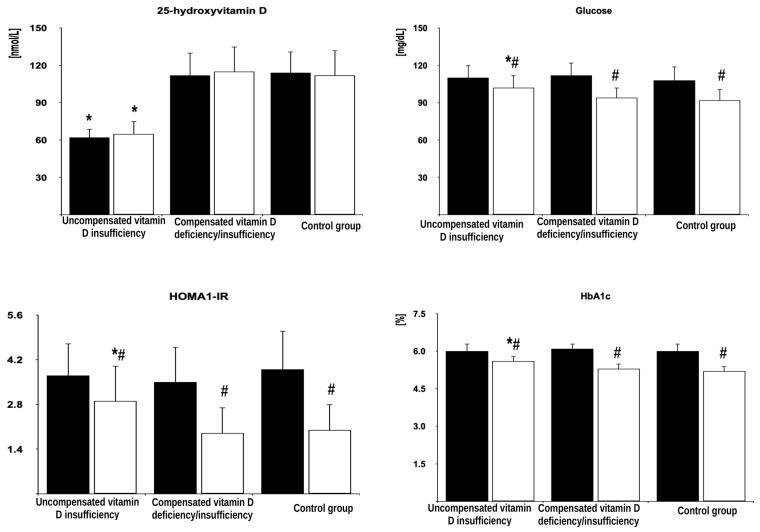
The effect of metformin on 25-hydroxyvitamin D levels and glucose homeostasis markers in postmenopausal women with hyperthyroidism and different vitamin D status. The data are shown as the mean ± standard deviation. Black bars: concentration before metformin treatment; white bars: concentration after metformin treatment. * *p* < 0.05 vs. values at the same time point in the remaining two groups. ^#^ *p* < 0.05 vs. baseline values in the same study group.

**Figure 3:**
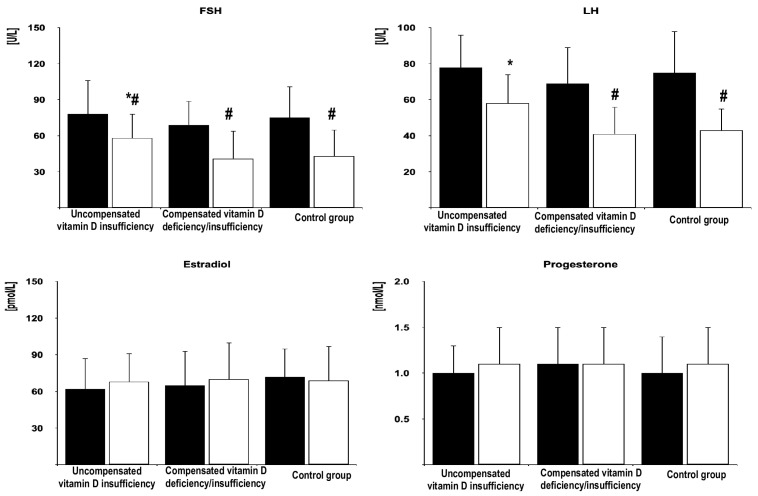
The effect of metformin on levels of gonadotropins, estradiol, and progesterone in postmenopausal women with hyperthyroidism and different vitamin D status. The data are shown as the mean ± standard deviation. Black bars: concentration before metformin treatment; white bars: concentration after metformin treatment. * *p* < 0.05 vs. values at the same time point in the remaining two groups. ^#^ *p* < 0.05 vs. baseline values in the same study group.

**Figure 4:**
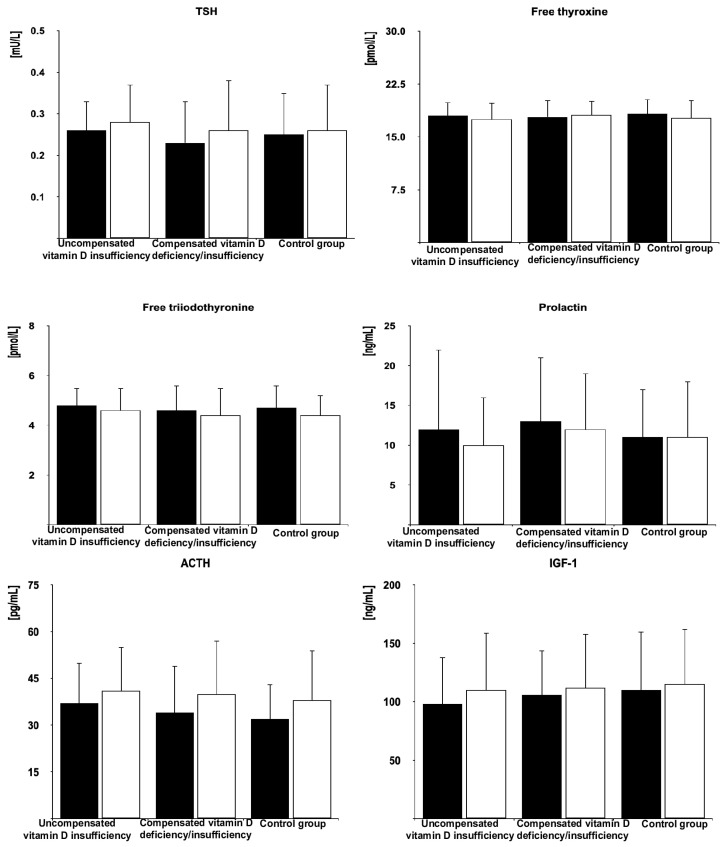
The effect of metformin on plasma levels of the remaining hormones in postmenopausal women with hyperthyroidism and different vitamin D status. The data are shown as the mean ± standard deviation. Black bars: concentration before metformin treatment; white bars: concentration after metformin treatment.

In all the groups of women after menopause, metformin treatment reduced fasting glucose, HOMA-IR, HbA_1_, and FSH. Moreover, the drug decreased LH levels in women with compensated vitamin D deficiency/insufficiency and in the control group but not in patients with uncompensated vitamin D insufficiency. Metformin treatment did not affect circulating levels of 25-hydroxyvitamin D, estradiol, progesterone, TSH, free thyroxine, free triiodothyronine, prolactin, ACTH, and IGF-1. The follow-up values of glucose, HOMA-IR, HbA_1_, FSH, and LH were higher in women with uncompensated vitamin D insufficiency than in the remaining groups but did not differ between women with compensated vitamin D deficiency/insufficiency and the control group (Figure 2, Figure 3 and Figure 4 and Supplementary Table S1).

### 3.4. Between-Group Comparisons

The percentage changes in glucose, HOMA1-IR, HbA_1c_, FSH, and LH were more pronounced in women with compensated vitamin D deficiency/insufficiency and in the control group than in patients with uncompensated vitamin D insufficiency but did not differ between both groups of women with normal vitamin D status (Table 2).

### 3.5. Correlations

Treatment-induced changes in FSH and LH concentrations positively correlated with baseline concentrations of (a) gonadotropins (*r* values between 0.461 [*p* = 0.0002] and 0.512 [*p* < 0.0001] for FSH and between 0.438 [*p* = 0.0008] and 0.484 [*p* = 0.0001] for LH), (b) free thyroxine (*r* values between 0.387 [*p* = 0.0023] and 0.456 [*p* = 0.004] for FSH and between 0.375 [*p* = 0.0042] and 0.410 [*p* = 0.0014] for LH), (c) free triiodothyronine (*r* values between 0.320 [*p* = 0.0295] and 0.368 [*p* = 0.0056] for FSH and between 0.295 [*p* = 0.0425] and 0.364 [*p* = 0.0051] for LH), and (d) 25-hydroxyvitamin D (*r* values between 0.415 [*p* = 0.0012] and 0.471 [*p* = 0.0002] for FSH and between 0.426 [*p* = 0.0010] and 0.460 [*p* = 0.0007] for LH). Moreover, there were positive correlations between the impact of metformin on gonadotropin levels and HOMA1-IR (*r* values between 0.288 [*p* = 0.0488] and 0.341 [*p* = 0.0204] for FSH and between 0.318 [*p* = 0.0298] and 0.357 [*p* = 0.0086] for LH). Treatment-induced changes in FSH and LH did not correlate with vitamin D intake with food, vitamin D intake with tablets, and supplementation duration.

## 4. Discussion

Irrespective of the group assignment, metformin did not have an impact on concentrations of TSH and free thyroid hormones. Moreover, there were no patients who prematurely terminated this study because of a worsening of thyroid function, or patients who completed this study despite deterioration of hyperthyroidism. A neutral effect on hypothalamic–pituitary–thyroid axis activity in hyperthyroid women, though contrasted with a decrease in TSH concentrations in individuals with elevated TSH concentration [1,2], was in line with our recent study including postmenopausal women with hyperthyroidism [17]. However, unlike that study, the participants of the current one were only patients with thyroid hyperfunction of non-autoimmune origin. In patients (mainly women) with autoimmune thyroiditis, metformin was found to decrease thyroid peroxidase and thyroglobulin antibodies [34]. If the drug had a similar effect on thyroid receptor antibodies in patients with Graves’ disease, it would affect the hypothalamic–pituitary–thyroid axis at the thyroid level, potentially masking a direct effect of metformin on TSH production. However, the current inclusion criteria eliminated such a possibility. The obtained results also allow us to conclude that vitamin D status does not determine the impact of metformin on the hypothalamic–pituitary–thyroid axis in patients with non-autoimmune thyroid hyperfunction.

In postmenopausal women with normal vitamin D homeostasis, metformin treatment caused a significant decrease in plasma levels of both FSH and LH. This effect was stronger than previously observed in women after menopause without thyroid pathology and with normal vitamin D status, in whom metformin action was limited to the changes in FSH levels [16]. These differences cannot be explained by baseline gonadotropin levels, which did not differ between participants of both studies. Moreover, considering the matching procedure and the exclusion criteria, they cannot be associated with the impact of comorbidities or comedications. Thus, the most probable explanation is the impact of concurrent hyperthyroidism, which is supported by positive correlations between the changes in FSH and LH concentrations and concentrations of free thyroid hormones.

A significant effect of metformin on gonadotropin secretion in postmenopausal women with hyperthyroidism seems to be clinically relevant because of detrimental effects of both thyroid hormone excess and elevated gonadotropin levels on cognitive and behavioral functions and on bone loss. Even subclinical hyperthyroidism was documented to predispose to dementia, low bone density, and osteoporotic fractures [35,36]. In turn, both FSH and LH were found to play a more important role in the pathogenesis of Alzheimer’s disease than low estrogen status [37,38]. Lastly, the rise of FSH in the late perimenopause may account, at least in part, for the rapid bone loss [39]. These findings suggest that the effects of gonadotropin excess and mild thyroid hyperfunction are, at least theoretically, additive and that the risk of cognitive decline and osteoporotic complications may be higher in case of their coexistence. Thus, the reduction in gonadotropin levels in the former group of women may be warranted, particularly if hyperthyroidism remains untreated. The obtained results may partially explain why long-term metformin therapy was associated with a lower incidence of neurodegenerative disorders [40], with a reduction in the risk of osteoporosis and osteoporotic fractures [41], and with an increase in bone mass density [41].

The impact of hyperthyroidism on the gonadotropin-lowering effect of metformin depended on the patients’ vitamin D status and was more pronounced in women with normal calciferol homeostasis than in women with vitamin D insufficiency. In the latter group, this effect was limited to a small decrease in FSH concentrations. Consequently, follow-up gonadotropin concentrations were higher in women with vitamin D insufficiency than in the remaining groups of women. It should be underlined that the interaction between thyroid hyperfunction and low vitamin D status was observed despite the fact that due to ethical concerns vitamin D-deficient women were not considered for enrollment if they had not been receiving calciferol supplementation. Thus, the attenuating effect on metformin action might have been even more pronounced in the case of patients with 25-hydroxyvitamin D below 50 nmol/L.

The molecular mechanisms involved in the interaction between vitamin D insufficiency and hyperthyroidism remain obscure. No statistically significant changes in circulating levels of 25-hydroxyvitamin D and thyroid hormones levels during this study indicate that they do not seem to mediate the gonadotropin-lowering effect of metformin. There is no evidence on the association between our findings and the impact on gluconeogenesis and the carrier 1 of organic cations, playing an important role in metformin action [42]. However, there are several important arguments suggesting that additive effects of vitamin D and hyperthyroidism on metformin action are secondary to interaction between vitamin D and thyroid hormone excess at the level of the pituitary 5′-adenosine monophosphate-activated protein kinase (AMPK) pathway. Firstly, this pathway is considered one of the most important mediators of metformin action [42]. Secondly, the pituitary gland, which is not protected by the blood–brain barrier [43], is able to concentrate significant amounts of metformin [44]. Thirdly, gonadotropic cells are characterized by the highest expression of AMPK among all types of anterior pituitary cells and were found to mediate metformin action on both baseline and stimulated gonadotropin secretion by rodent primary pituitary cells [45]. Fourthly, the activity of this pathway was found to be decreased in animals fed with a vitamin D-insufficient diet [46] and increased in experimentally induced hyperthyroidism [47]. Moreover, both exogenous vitamin D [48] and exogenous triiodothyronine [49] were reported to activate the AMPK pathway in peripheral tissues. Fifthly, activation of this pathway mediates the additive effects of calciferol and metformin on growth of prostatic cancer [50]. Lastly, this explanation well explains why differences in the strength of the gonadotropin-lowering effect of metformin were paralleled by similar differences in the impact on insulin sensitivity, the latter of which is partially associated with metformin-induced activation of AMPK [42].

Another interesting observation was the lack of differences in the pituitary effects of metformin between both groups of postmenopausal hyperthyroid women with normal vitamin D status. There were also no statistically significant differences between these groups in daily vitamin D intake with food. Lastly, daily vitamin D dose and duration of calciferol supplementation in women with previous vitamin D deficiency/insufficiency did not correlate with metformin-induced changes in gonadotropin concentrations. These findings indicate that differences in the strength of metformin action are not associated with the pharmacological action of exogenous calciferol and/or with pharmacokinetic interactions between exogenous vitamin D and TSH and/or thyroid hormones. What is important is that the inhibitory effect of low calciferol status on the pituitary effects of metformin in hyperthyroid women is not irreversible. A normal secretory response to metformin treatment may be restored by the normalization of vitamin D homeostasis. Considering that high levels of FSH and LH levels are an inevitable consequence of menopause, and may have negative health consequences, calciferol supplementation in women with low vitamin D levels may be important not only to prevent osteoporosis, particularly if they are simultaneously treated with metformin. Thus, our observations may justify the rationale of routine assessment of 25-hydroxyvitamin D in order to choose an appropriate dose of this vitamin for supplementation.

Despite reducing FSH and LH concentrations and between-group differences in this effect, metformin produced a neutral effect on estradiol levels, even in women with normal calciferol homeostasis. Moreover, there were no differences in follow-up values of this hormone between women with normal and subnormal 25-hydroxyvitamin D levels. As well, the gonadotropin-lowering effects of metformin did not correlate with the impact of metformin on estradiol. These findings agree that changes in gonadotropin concentrations, irrespective of vitamin D status, are not secondary to the improvement in ovarian secretory function or to increased estrogen production by extra-ovarian tissues (particularly the liver, adipose tissue, mammary glands, muscles, and skin) [51]. This putative selective effect on the hypothalamic–pituitary–ovarian axis limited to the pituitary hormones is intriguing and worth further investigation in light of controversies associated with hormone replacement therapy after menopause [52]. Similarly, calciferol homeostasis did not determine metformin action on circulating progesterone, the levels of which were low but stable over the entire study period, reflecting reduced and gonadotropin-independent production of this hormone by postmenopausal ovaries and the lack of association between its levels and gonadotropin secretion in middle-aged and elderly women [10,53]. It should be, however, mentioned that we did not measure estrone, the dominant estrogen after menopause [54]. However, owing to its much weaker estrogenic properties and only slightly higher levels in comparison with estradiol [54], it is difficult to assume that between-group differences in estrone concentrations, if at all exist, may contribute to the observed differences in response of gonadotropic cells to chronic metformin administration.

The study results allow us to also draw other conclusions that are important from the practical point of view. Firstly, despite high-dose treatment, metformin was well tolerated, and its adverse effects were reported only in a minority of patients. Hence, this treatment appears to be safe in middle-aged and elderly women with prediabetes and concurrent thyroid hyperfunction. Secondly, vitamin D status had a neutral effect on metformin action on ACTH and IGF-1 in hyperthyroid women, which can be explained by the fact that their source was anterior pituitary cells with normal resting secretory activity. Furthermore, no cases of subnormal pituitary hormone levels indicate that high-dose metformin treatment does not carry the risk of iatrogenic pituitary hypofunction. Lastly, normal vitamin D status is required to maximize the insulin-sensitizing effect of metformin in prediabetic women and mildly increased thyroid function. It is worth mentioning that hyperthyroidism by itself seems to predispose to type 2 diabetes [55]. However, the restoration of normal calciferol homeostasis may delay or even prevent the development of type 2 diabetes in hyperthyroid women after menopause at high diabetes risk who are receiving metformin.

Several shortcomings of this study should be considered when interpreting our findings. A moderate sample size increased the risk of statistical type II errors (failing to reject a false null hypothesis). Due to the cohort nature, the study results might have been influenced by selection, information, and confusion biases. It is difficult to draw strong conclusions on interactions between more severe disturbances in vitamin D homeostasis and more severe stages of hyperthyroidism or hyperthyroidism of autoimmune origin because women with 25-hydroxyvitamin D levels below 50 nmol/L, grade 2 subclinical hyperthyroidism, overt hyperthyroidism, and Graves’ disease were not considered eligible for this study. Extrapolating previous data [56,57], it seems that the participants were characterized by adequate iodine consumption and by selenium deficiency. It remains to be elucidated whether pituitary effects of metformin in postmenopausal women with low vitamin D status and thyroid hyperfunction are the same in the case of inadequate iodine and/or sufficient selenium intake. We cannot completely rule out a late-appearing effect of non-pharmacological treatment. Lastly, this study does not provide insight into the molecular mechanisms underlying the interaction between vitamin D insufficiency and hyperthyroidism.

## 5. Conclusions

The effect of metformin on gonadotropin levels in postmenopausal women with hyperthyroidism was stronger if they were characterized by normal calciferol homeostasis than low vitamin D status. The interaction between thyroid hormone excess and vitamin D homeostasis on gonadotropin production in response to metformin treatment was likely mediated by the impact on the AMPK pathway in pituitary gonadotropic cells. The effect on gonadotropin secretion was not accompanied by changes in estradiol and progesterone production but was paralleled by similar differences in the impact on insulin sensitivity. Despite thyroid hyperfunction, irrespective of calciferol status, chronic metformin treatment did not affect hypothalamic–pituitary–thyroid axis activity. Our findings suggest that low vitamin D status attenuates the gonadotropin-lowering effects of metformin in individuals with hyperthyroidism and that even mild disturbances in calciferol homeostasis in postmenopausal women receiving metformin need to be adequately supplemented. Because of this study’s limitations, our preliminary findings require replication and validation in larger studies.

## Figures and Tables

**Table 1:** Baseline characteristics of women participating in this study and baseline levels of the investigated variables.

Variable	Uncompensated Vitamin D Insufficiency	Compensated Vitamin D Deficiency/Insufficiency	Control Group
Number (n)	23	22	24
Age (years)	65 ± 8	64 ± 9	65 ± 7
Multinodular goiter/solitary adenomas (%)	74/26	73/23	75/25
Smokers (%)/number of cigarettes a day (n)/duration of smoking (years)	43/9 ± 6/30 ± 11	45/9 ± 5/31 ± 14	38/10 ± 6/32 ± 12
Body mass index (kg/m^2^)	24.6 ± 4.7	23.8 ± 4.9	23.6 ± 5.1
Systolic blood pressure (mmHg)	130 ± 17	128 ± 16	127 ± 15
Diastolic blood pressure (mmHg)	83 ± 6	81 ± 5	81 ± 6
Daily calciferol intake with food ^1^ (µg)	10.0 ± 5.5	9.5 ± 5.0	11.3 ± 6.0
25-hydroxyvitamin D (nmol/L)	62 ± 7 *	112 ± 18	114 ± 17
Glucose (mg/dL)	110 ± 10	112 ± 10	108 ± 11
HOMA1-IR	3.7 ± 1.0	3.5 ± 1.1	3.9 ± 1.2
HbA_1c_ (%)	6.0 ± 0.3	6.1 ± 0.2	6.0 ± 0.3
FSH (U/L)	78 ± 28	69 ± 20	75 ± 26
LH (U/L)	48 ± 18	44 ± 20	50 ± 23
Estradiol (pmol/L)	62 ± 25	65 ± 28	72 ± 23
Progesterone (nmol/L)	1.0 ± 0.3	1.1 ± 0.4	1.0± 0.4
TSH (mU/L)	0.26 ± 0.07	0.23 ±0.10	0.25 ± 0.10
Free thyroxine (pmol/L)	18.0 ± 1.9	17.8 ± 2.4	18.3 ± 2.0
Free triiodothyronine (pmol/L)	4.8 ± 0.7	4.6 ± 1.0	4.7 ± 0.9
Prolactin (ng/mL)	12 ± 10	13 ± 8	11 ± 6
ACTH (pg/mL)	37 ± 13	34 ± 15	32 ± 11
IGF-1 (ng/mL)	98 ± 40	106 ± 38	110 ± 50

Unless otherwise stated, the data are shown as the mean ± standard deviation. ^1^ without vitamin D contained in tablets. * *p* < 0.05 vs. values in the remaining two groups.

**Table 2:** Percentage changes from baseline in the investigated variables in postmenopausal women with hyperthyroidism and different vitamin D status.

Variable	Uncompensated Vitamin D Insufficiency	Compensated Vitamin D Deficiency/Insufficiency	Control Group
Δ25-hydroxyvitamin D	5 ± 15	3 ± 11	−2 ± 14
ΔGlucose	−7 ± 8 *	−16 ± 10	−15 ± 9
ΔHOMA1-IR	−22 ± 25 *	−46 ± 31	−49 ± 32
ΔHbA_1c_	−7 ± 8 *	−13 ± 10	−13 ± 9
ΔFSH	−26 ± 20 *	−41 ± 22	−43 ± 25
ΔLH	−17 ± 25 *	−46 ± 26	−38 ± 21
ΔEstradiol	10 ± 29	8 ± 32	−4 ± 29
ΔProgesterone	10 ± 50	0 ± 35	10 ± 42
ΔTSH	8 ± 15	13 ± 19	4 ± 20
ΔFree thyroxine	−3 ± 8	−4 ± 6	−3 ± 7
ΔFree triiodothyronine	−4 ± 10	−4 ± 8	−6 ± 7
ΔProlactin	−17 ± 30	−8 ± 28	0 ± 35
ΔACTH	11 ± 20	18 ± 23	19 ± 25
ΔIGF-1	12 ± 15	6 ± 19	5 ± 17

The data are shown as the mean ± standard deviation. * *p* < 0.05 vs. percentage changes from baseline in the remaining two groups.

## Data Availability

The original contributions presented in this study are included in the article. Further inquiries can be directed to the corresponding author.

## Supplementary Materials

The following supporting information can be downloaded at https://www.mdpi.com/article/10.3390/pharmaceutics17040442/s1, Table S1. The effect of metformin on the investigated variables in postmenopausal women with hyperthyroidism and different vitamin D status.
