# Supplementary material for: Vitamin D Status Determines Metformin Action on Gonadotropin Levels in Postmenopausal Women with Subclinical Hyperthyroidism
Source: Pharmaceutics. 2025 Mar 30;17(4):442. doi: 10.3390/pharmaceutics17040442 (PMC12030551; doi:10.3390/pharmaceutics17040442)
Supplement: Supplementary file 1 [file pharmaceutics-17-00442-s001.zip › pharmaceutics-3522355-supplementary.pdf]

Table S1. The effect of metformin on the investigated variables in postmenopausal women with and hyperthyroidism and different vitamin D status.

Variable	Uncompensated vitamin D insufficiency	Compensated vitamin D deficiency/insufficiency	Control group
**25-hydroxyvitamin D** (nmol/L)			
Before metformin treatment	62±7*	112±18	114±17
After metformin treatment	65±10*	115±20	112±20
**Glucose** (mg/dL)			
Before metformin treatment	110 ± 10	112 ± 10	108±11
After metformin treatment	102 ± 10*#	94 ± 8#	92 ± 9#
**HOMA1-IR**			
Before metformin treatment	3.7±1.0	3.5±1.1	3.9±1.2
After metformin treatment	2.9±1.1*#	1.9±0.8#	2.0±0.8#
**HbA_{1c}** (%)			
Before metformin treatment	6.0±0.3	6.1±0.2	6.0±0.3
After metformin treatment	5.6±0.2*#	5.3±0.2#	5.2±0.2#
**FSH** (U/L)			
Before metformin treatment	78 ± 28	69 ± 20	75 ± 26
After metformin treatment	58 ± 20*#	41 ± 23#	43 ± 22#
**LH** (U/L)			
Before metformin treatment	48 ± 18	44 ± 20	50 ± 23
After metformin treatment	40 ± 16*	30 ± 15#	31 ± 12#
**Estradiol** (pmol/L)			
Before metformin treatment	62 ± 25	65 ± 28	72 ± 23
After metformin treatment	68 ± 23	70 ± 30	69 ± 28
**Progesterone** (nmol/L)			
Before metformin treatment	1.0 ± 0.3	1.1 ± 0.4	1.0± 0.4
After metformin treatment	1.1 ± 0.4	1.1 ± 0.4	1.1 ± 0.4
**TSH** (mU/L)			
Before metformin treatment	0.26±0.07	0.23±0.10	0.25±0.10
After metformin treatment	0.28±0.09	0.26±0.12	0.26±0.11
**Free thyroxine** (pmol/L)			
Before metformin treatment	18.0±1.9	17.8±2.4	18.3±2.0
After metformin treatment	17.5±2.3	17.1±2.0	17.7±2.5
**Free triiodothyronine** (pmol/L)			
Before metformin treatment	4.8±0.7	4.6±1.0	4.7±0.9
After metformin treatment	4.6±0.9	4.4±1.1	4.4±0.8
**Prolactin** (ng/mL)			
Before metformin treatment	12 ± 10	13 ± 8	11 ± 6
After metformin treatment	10 ± 6	12 ± 7	11 ± 7
**ACTH** (pg/mL)			
Before metformin treatment	37 ± 13	34 ± 15	32 ± 11
After metformin treatment	41 ± 14	40 ± 17	38 ± 16
**IGF-1** (ng/mL)			
Before metformin treatment	98 ± 40	106 ± 38	110 ± 50
After metformin treatment	110 ± 49	112 ± 46	115 ± 47

The data are shown as the mean ± standard deviation.

* $p < 0.05$ vs. values at the same time point in the remaining two groups. # $p < 0.05$ vs. values before metformin treatment in the same study group.
